# Internet of things (IoT) based saffron cultivation system in greenhouse

**DOI:** 10.1038/s41598-024-69513-1

**Published:** 2024-09-29

**Authors:** Rabia Khan, Muhammad Shoaib Farooq, Adel Khelifi, Umer Ahmad, Faizan Ahmad, Shamyla Riaz

**Affiliations:** 1 Scool of System and Technology, University of Management and Technology, Lahore, 54000 Pakistan; 2 Computer Science and Information Technology, Abu Dhabi University, Abu Dhabi, United Arab Emirates; 3 Department of Computer Science, Garrison University Lahore, Lahore, Pakistan; 4 Cardiff School of Technologies, Cardiff Metropolitan University, Cardiff, UK

**Keywords:** Internet of things, Greenhouse, IoT sensors, Saffron, Agronomical factors, Architecture, Natural variation in plants, Plant evolution, Computer science, Information technology

## Abstract

Saffron is the world's most expensive and legendary crop that is widely used in cuisine, drugs, and cosmetics. Therefore, the demand for saffron is increasing globally day by day. Despite its massive demand the cultivation of saffron has dramatically decreased and grown in only a few countries. Saffron is an environment-sensitive crop that is affected by various factors including rapid change in climate, light intensity, pH level, soil moisture, salinity level, and inappropriate cultivation techniques. It is not possible to control many of these environmental factors in traditional farming. Although, many innovative technologies like Artificial Intelligence and Internet of Things (IoT) have been used to enhance the growth of saffron still, there is a dire need for a system that can overcome primary issues related to saffron growth. In this research, we have proposed an IoT-based system for the greenhouse to control the numerous agronomical variables such as corm size, temperature, humidity, pH level, soil moisture, salinity, and water availability. The proposed architecture monitors and controls environmental factors automatically and sends real-time data from the greenhouse to the microcontroller. The sensed values of various agronomical variables are compared with threshold values and saved at cloud for sending to the farm owner for efficient management. The experiment results reveal that the proposed system is capable to maximize saffron production in the greenhouse by controlling environmental factors as per crop needs.

## Introduction

Agriculture is the oldest industry in the world^1^ which plays an essential role in stabilizing the economy of any country. Due to deforestation and dramatic climate change, this industry was badly affected^2^. Latest advancements in technology and machinery have brought a revolution in this industry to meet the food requirements globally. Recently Internet of things (IoT)^3^, computer vision, remote sensing and Artificial Intelligence^4^, machine learning^5^, and wireless sensor networks^6,7^ have shown promising results in the agriculture industry by remotely controlling and monitoring the fields, harvesting through robots^8,9^, and automatic irrigation^10^ and pest control system. These technologies are broadly used in the early detection and prediction of crop health^11^ and their growth pattern^9^ over a period of time by storing filed data hence contributing to sustainable agriculture^12^. The inclusion of IoT technologies in agriculture is not only contributing to optimizing crop growth but also reducing labor costs and efficient resource management^13^. IoT technologies have the capability to store huge amounts of data in a secure environment^14,15^ for analysis and disease prediction^16^. Keen analysis of stored data could also be used to find the relationship between different environmental variables such as soil characteristics, and climate variables including temperature, humidity, rainfall^17^ light intensity etc.^13^ on the basis of which advance planning can be done to mitigate the future risks by opting different. There are numerous crops around the world that are climate sensitive and cannot be grown in every region of the world due to unfavorable circumstances e.g. mushrooms, olives, Soybean, and saffron. For such kinds of crops inclusion of technology is a must in order to achieve optimal results while controlling environmental factors.

**Figure 1:**
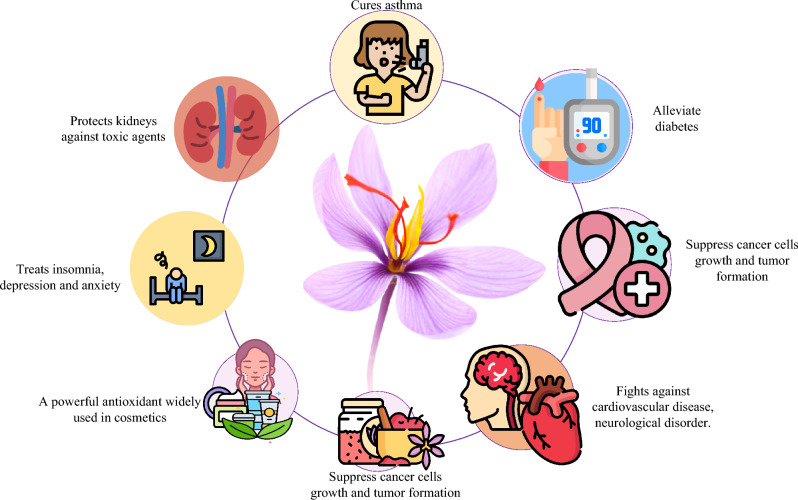
Benefits of Saffron in cosmetology, health and food industry.

Saffron is the world’s oldest, most expensive, and legendary crop. Few countries are only famous for the huge cultivation of this golden spice including Iran, Italy, Spain, Greece, India, and Afghanistan^18^. Saffron is known for its unbeatable aroma, taste food flavor, and for several applications in the healthcare sector due to extravagant advantages in cuisine, drugs, and beauty products^19^. In the modern era scientist are searching for medicinal plants because of their minimal side effects^20^. Among different medicinal plants saffron is considered one of the most effective herbs. Saffron has a historical background in medicine due to its positive impact on human health. This crop plays a vital role in treating several diseases including cardiovascular^21,22^, asthma^23^, suppressing cancer growth^21,24^, and cell repairing^25^. Furthermore, it is also ideal for stomach diseases including Gastrointestinal, abdominal pain^26^, neurodegeneration^27^, age-related diseases^28^, ocular diseases^29^, and eye disease^30,31^. There is a sudden decrease in saffron growth globally due to various factors including soil acidification, drastic climate change, temperature, light intensity, fertilization, excessive use of water, and inappropriate cultivation techniques^18^. Traditional farming cannot control these factors appropriately. Figure 1 shows the most common use of saffron in different areas.

In the past few years, Greenhouses caught attention of the many researchers and farmers around the globe. The main reason behind the popularity of Greenhouse among farmers and researchers is when it comes to growing off-season and environment-sensitive crops or vegetables. Greenhouse provides a controlled environment to the farmers where they can make alterations in the artificial environment according to the needs of several crops and vegetables to maximize their growth^32^. Greenhouses are playing a crucial role in overcoming the food shortage and contributing to achieving Sustainable Development Goal 2^33^. Most famous greenhouse crops globally include mushrooms, tomatoes, peppers, cucumber, and saffron^34,35^. Saffron is among those crops, that are quickly affected by climate change, and in traditional farming, it is not possible to control climate in a selective region of the world. Various techniques are suggested by the researchers to increase the yield of various crops using IoT systems in the greenhouse including mushrooms^36^, tomatoes^37^, and cucumbers^38^.

Similarly, many researchers have proposed several techniques to optimize saffron growth. Many of these proposed techniques were based on field study and mainly focused on the cause of deterioration^39^, saffron quality, suitable soil conditions^40^, planting dates management^41^, temperature effects^42^, and land suitability models for saffron^43^, etc. In the realm of saffron cultivation research, despite the exploration of various techniques, the declining saffron yield persists due to unmanageable factors. The most common factors include climate change, erratic temperature variations, fluctuating light intensity, soil moisture levels, salinity, pH imbalances, and unpredictable availability of water in traditional farming practices.

The primary focus of this research article is to maximize the yield of saffron globally. To achieve this goal most essential agronomical variables playing a vital role in saffron growth were figured out from the literature that cannot be controlled in traditional framing. In this research, we have proposed a new IOT system for the Greenhouse which is capable of monitoring and controlling environmental factors automatically affecting saffron growth and maximizing the yield of saffron. The new proposed system is capable of sensing real-time data from the greenhouse through the sensor and sending it to the microcontroller. Sensed values of various agronomical variables including temperature, humidity, soil salinity, pH level, and soil moisture will be compared with threshold values saved in the cloud for sending appropriate messages to the farm owner for efficient management. An appropriate platform for monitoring and controlling the saffron growth has been provided to the farmer. The experiment results reveal that the proposed model is capable of maximizing saffron production in the greenhouse where we can control the environmental factors as per saffron needs.

The rest of the article is divided into the following sections: In “Work flow” related work done in the past is discussed along with accomplishments and limitations. “Related work” shows the proposed systems in detail and in “Materials and Methods” proposed methodology is described. Finally last but not the least “Conclusion” presents the conclusion of this article.

## Work flow

We have developed an IoT-based system for saffron cultivation that includes numerous stages. In the first stage, an extensive literature review is conducted to identify different agronomical variables crucial for saffron. These variables include temperature, humidity, soil moisture, soil salinity, corm size, and light intensity along with their optimal ranges. Later on, we selected a suitable sensor for each agronomical variable.

Moving on to the sensor selection, we establish a greenhouse setup. In the initial stage, a greenhouse chamber was chosen along with loamy soil due to its suitability for saffron. A load cell sensor was placed within the greenhouse chamber but outside the farming area, in a dry place, to determine the corm size. Furthermore, the actuators including the water pump and LED lights were placed at the center of the greenhouse for optimal coverage in every direction. Temperature, humidity, and LDR sensors were positioned 40–50 cm above the saffron field to capture desired environmental factors. Ground-based sensors were inserted into the soil at the depth of 30–40 cm for measuring soil salinity, moisture, and pH.

Once all sensors were in their positions, they all were connected to microcontrollers and relays. For monitoring purposes, real-time data was transmitted to the microcontroller, which then transmitted this information to the Blynk IoT app where users could log in using their credentials making sure that only authenticated users could access the data. Farm owners could analyze the field conditions by using this platform.

## Related work

Researchers and agriculturists have been doing research for decades in the agriculture domain to enhance crop productivity and overcome food shortages. After doing a lot of research in the agriculture field, researchers came to know about the decrement in the yield of saffron. To increase the productivity of the saffron crop different techniques have been used by implementing Machine learning and IoT technology.

Anosheh et al.^41^ investigated the feasibility of saffron cultivation by using semi-saline water and found that early planting led to a higher yield and better quality of saffron. Managing the planting date can be a practical solution for saffron cultivation by using semi-saline water that provides valuable insights for saffron farmers. Although the proposed solution is best for effective saffron cultivation the potential environmental impact of using semi-saline water for saffron production was not explored in the long term.

Molina et al.^42^ discussed the impact of temperature on saffron growth and found that low temperatures affect negatively during corm development. Furthermore, high temperatures during flower bud initiation also influence the yield of saffron badly. Moreover, different strategies have been discussed to control the effect of the temperature on saffron growth that helps the growers to optimize cultivation techniques.

Maleki et al.^43^ developed a model to identify suitable land for saffron cultivation by integrating several factors such as soil, climate, and topography by using a multi-criteria evaluation approach. The results showed that well-drained calcareous soil, a warm and dry climate, and an altitude range of 800–1800 m are most suitable for saffron cultivation. The study highlights the importance of MCE and GIS in land suitability analysis and provides valuable insights for farmers and policymakers for saffron cultivation. However, this research only focuses on finding suitable land in a specific area of Iran that may not be suitable for other regions due to variations in climate and type of land.

Daneshmandi et al.^44^ have investigated the effect of using composted pistachio residues and commercial poultry manure as organic amendments on nutrient availability and saffron corm growth in calcareous soil. The research can help saffron growers optimize their soil fertility management practices and improve the quality and quantity of their saffron production. However, environmental factors such as climate and crop management strategies were not taken into consideration. Furthermore, the soil used for the greenhouse was taken from a non-cultivate farm in Torbat Heydarieh, Iran. Therefore, the findings may not be suitable for the other regions.

Sepaskhah et al.^45^ determined the optimal amount of irrigation water and corm planting density for saffron cultivation in a semi-arid region. Traditional flood irrigation and drip irrigation methods were compared to identify the most effective approach for optimizing saffron yield and quality. The results can help saffron growers improve their production while reducing water usage and promoting sustainable cultivation practices. However, this research study does not consider other crucial factors playing a vital role in the growth of saffron including soil characteristics, temperature, light, and humidity. There were some references that were cited in the study in Persian language, which makes it difficult to evaluate the research findings.

Yarami et al.^46^ analyzed the impact of irrigation water salinity, manure application, and planting methods on the growth and gas exchange response of saffron. The study aims to identify the optimal levels of these factors for achieving the best growth and productivity in saffron crops. The findings can help saffron growers to optimize their cultivation practices while providing insights into how saffron plants respond to environmental stress factors.

The semi-aired region was taken into consideration for saffron production in this experiment so findings may not be suitable for other regions with different climate conditions.

The primary purpose of Koocheki et al.^47^ was to determine the optimal combination of nitrogen and phosphorus fertilization to improve crop yield. The findings of the study can help saffron growers to optimize their fertilizer application practices. However, this research was conducted at a specific location in Mashhad, Iran, so the findings may not be suitable for other regions with different types of climate and soil properties, also other factors which affect the yield of saffron are not discussed.

Esmaeilian et al.^48^ have discussed the effects of organic and biological fertilizers on saffron cultivation. The study indicates that these fertilizers can improve soil properties and nutrient availability, leading to higher saffron yield. However, the study was conducted in a specific region, and economic feasibility was not investigated. Data used for the experiment was hidden due to confidentially which is why it is not possible to validate the results of this study. Furthermore, the impact of biological and organic fertilizers used for saffron cultivation has not been discussed.

Saffron water requirements depend on several factors, including temperature, humidity, soil type, and plant growth stage. The water demand for saffron is highest during the vegetative stage and decreases during the flowering and dormant stages. Saffron requires well-drained soil and a consistent supply of moisture, but overwatering can lead to root rot and decreased yield.

Generally, saffron requires 350–500 mm of water per year, with irrigation requirements varying on the local climate and soil conditions. Koocheki et al.^49^ discussed that accurate water management is critical for optimal saffron production, and using efficient irrigation methods can help minimize water use while maximizing crop yield. Although, water-related aspects were the primary focus of the research soil and climate conditions have not been considered which are highly important for saffron cultivation.

The potential benefits of shifting saffron cultivation from traditional open-field farming to greenhouse production have been discussed by Khorasgani et al.^50^. The authors argue that saffron is vulnerable to climate change, and growing it in a controlled environment can improve productivity and quality. The study also discusses factors involved in the greenhouse production of saffron, such as light intensity, temperature, humidity, and nutrient management. However, no automated strategies for controlling and monitoring the greenhouse were mentioned in this research. Furthermore, Table 1 presents the challenges and limitations of already available solutions for saffron crops.

**Table 1:** Challenges and limitations in current studies.

Sr. no.	Author	Work done	Crop	Results	Limitations
^51^	Salas et al	The main objective of this research was to enhance saffron corm production in the soilless greenhouse by optimizing nutrient solutions. Three different levels of Electro conductivity (EC) were selected for this experiment 2.0, 2.5, and 3.0, and only one type of substratum	Saffron	The results of the conducted experiment have shown that the highest concentration of nutrients was at a higher level of EC which is 3.0. Increased nutrient uptake produces best quality corms which enabled a 3 to 5 times increase in saffron yield	Only three levels of EC ranging from 2.0 to 3.0 were considered with a single type of substratum for soilless cultivation of saffron which may not be applicable for other EC levels with different types of perlite. Moreover, other essential environmental factors that could play a significant role in optimal saffron cultivation are not investigated
^52^	Kumar et al	Ecological Niche Modelling (ENM) using MaxEnt was used to identify the suitable areas for saffron cultivation in India on the basis of environmental parameters and terrain	Saffron (Crocus sativus)	Six new regions in India were found suitable for saffron cultivation. Temperature and rainfall were considered the most important environmental factors affecting saffron growth. The yield and quality of saffron produced in new regions was compared with traditional grown results depicting that saffron produced in new regions is as good as that produced in traditional grown	The developed model considers only the global environmental variables and sparse presence data neglecting local variations and specific requirements of saffron with respect to land
^53^	Stelluti et al	The effects of beneficial micro-organisms on the quality of saffron were investigated in this article	saffron (*Crocus sativus* L.)	The content of safranal was enhanced by 96% in treated plants but did not improve flower and spice yield significantly. Total phenolic content was increased by 19% with the help of mixed treatments of AMF and PGPR. Mixed treatment also restored corm size and increased the weight by 24%	The primary objective of this research was not to increase the yield but quality enhancement
^54^	Shajari et al	A field experiment was conducted at the University of Birjand for two growing seasons to improve saffron flower corm production. Factorial based on randomized complete block design with two experimental factors namely soil texture and irrigation intervals are used in this research. Effects of temperature, rainfall, and corm size were also studied during the experiment. Soil textures and their physiological properties were evaluated to see their effect on saffron yield	saffron (*Crocus sativus* L.)	The results of this research concluded that soil with moderate soil texture and with a one-week irrigation interval enhanced the corm and flower growth. Stigma and corm yield was increased in soil with a higher sand ratio. Whereas the soil with heavy texture and with two weeks of irrigation interval reduced the flower and corm yield	Irrigation management strategies being used in this research were discussed. Moreover experiment was conducted in small pots due to which the concluded results may not be suitable for larger fields
^55^	Moshizi et al	For the prediction of green and blue water footprint, a new optimized GMDH and ensemble model is developed for saffron crops. The optimized model consists of three stages and at each stage different algorithms are being used. The first stage is comprised of the GMDH model along with evolutionary algorithms to evaluate the individual inputs of the most effective variables of water footprints. IMM is applied in the second stage where outputs of the previous stage are incorporated as inputs of this stage to predict the green and blue water footprints for the saffron crop. Lastly, the uncertainty of this optimized model is evaluated on the basis of input parameters	Saffron	A new optimized prediction model for green and blue water footprints for saffron crops has been developed. The most crucial variables affecting water footprints were determined including, yield, plant transpiration, and water loss by removing these factors RMSE can be increased. NMRA algorithm of GMDH outperformed in this research due to high accuracy and quick convergence rate. Whereas IMM has lower uncertainty with better performance	Scenarios considered for this research are not publically available. Moreover, the criteria for selecting input parameters which was one of the most crucial phases are not discussed in detail
^56^	Cardone et al	Field study for the saffron crop is conducted for two years from 2017 to 2019 in Italy to compare three corm classes with dimensions (2.0–2.5 cm), (2.6–3.5 cm) and (3.6–4.5 cm) for two crops cycle including annual and biennial to evaluate the growth and quality of corms with different dimensions	Saffron	Higher stigma yield with quality was produced in the biennial crop cycle whereas, heavier and bigger flowers and corms were produced in the annual crop cycle in class three with corm dimension (3.6–4.5 cm). The highest no. of flowers and stigma were produced by corm of class two with dimensions (2.6–3.5 cm)	Only crop life cycle and corm dimension were evaluated the other crucial factors that have the potential to influence the saffron growth are not discussed such as irrigation, temperature, planting density, etc
^57^	Kothari et al	Field experiment for the years 2018–2019 and 2019–2020 was conducted at six different locations in the Western Himalayas for saffron cultivation. A factorial randomized block design was used to carry out this experiment. The main objective of this research was to check the suitability of different non- traditional locations for saffron crops based on environmental factors such as annual rainfall, humidity, temperature, and altitude	Saffron	The results show that environmental factors such as temperature, altitude, soil texture, humidity, and annual rainfall have the potential to influence the quality and growth of saffron. Location 3 of this research named Suppa Bharmour has produced the highest yield of with best quality as it has a high altitude, less humidity and rainfall, sandy loam soil texture, and optimal temperature ranging from 16.5 to 28.2 °C	The obtained results were not compared with traditional saffron growing areas of India, which may influence the saffron growth due to different soil conditions and climate than the non-traditional areas. Moreover, other important factors such as light intensity and irrigation techniques were not discussed
^58^	Chaouqi et al	This research study evaluates the impact of soil on secondary metabolites of saffron. Soil samples were analyzed with the help of ED-XRF fluorescence and X-ray diffraction methods to evaluate their physicochemical properties. Saffron samples obtained from soils with different properties were analyzed using the HPLC–DAD method for their quality evaluation	Moroccan Saffron (*Crocus sativus* L.)	Soil textures, potassium, phosphorus, and organic matter had potential influence on metabolites of saffron namely crocins, safranal, and kaempferol 3- sophrroside 7- glucoside. Clays with low levels of iron had a positive impact on the coloring strength of saffron	Other important environmental factors such as temperature, climate, corm size, etc. were not discussed that may effect the quality and yield of the saffron crop
^59^	Hashemi et al	This research aims to find the effect of nitrogen and salinity on *Crocus sativus* L. Three different nitrogen levels and four different salinities of irrigation water were investigated. A factorial experiment based on randomized design was used in this experiment. Soil samples used in this experiment were also tested to evaluate their physicochemical	*Crocus sativus* L	Higher levels of soil salinity negatively affect the ion balance and saffron growth. Nitrogen supply of (50 kg N ha-1) improved the salt tolerance of saffron crops. Results depicted that Nitrogen supply has the potential to mitigate the effects of soil salinity on saffron	Other crucial factors such as environmental factors and nutrients were not considered that may influence salt tolerance in saffron cultivation
^60^	Gao et al	LED luminaires were designed and fabricated with a water cooling system to avoid thermal power management issues for saffron cultivation in vertical farming. The designed luminaires have the capability not only to provide a sufficient amount of light but also to manage heat levels. Moreover, a numerical model was developed to evaluate the effect of water cooling heat on saffron corm. The performance and energy consumption of white and blue-red luminaires were also compared	Saffron	LED luminaires with effective thermal management were developed to provide a sufficient amount of light intensity for saffron cultivation. The water cooling system has a positive impact on saffron corm while controlling temperature. Additionally, the comparison analysis of LED luminaires is more energy efficient as compared to blue-red LEDs as these LEDs provide higher radiant performance	The proposed model is based on assumptions and interpretations that may not be able to present real-world conditions in open fields
Proposed model		Hence, the literature shows that different techniques have been proposed by the researchers to optimize saffron growth. But, still, there is a dire need for a system that can overcome primary issues related to saffron growth including light intensity, temperature, humidity, pH level, soil moisture level, irrigation, and soil salinity. To overcome these challenges this study investigates the most significant agronomical variable from the literature that cannot be managed in traditional open field farming. This research presents an IoT-based greenhouse architecture to monitor and control the agronomical variables automatically in a controlled environment	Saffron	The experiment results ensure that the proposed system has the potential to maximize the saffron yield in the greenhouse by controlling necessary environmental factors as per saffron crop needs	

## Materials and methods

An IoT-based architecture has been designed to optimize the growth of saffron crops in the greenhouse. The proposed system consists of multiple sensors namely a soil moisture sensor, Soil salinity sensor, Temperature and Humidity sensor, PH sensor, Light sensor, load cell sensor, a microcontroller, and an actuator. In this section, we have presented the hardware implementations, their configuration, block diagram, and proposed architecture.

### Hardware setup and design

**Figure 2:**
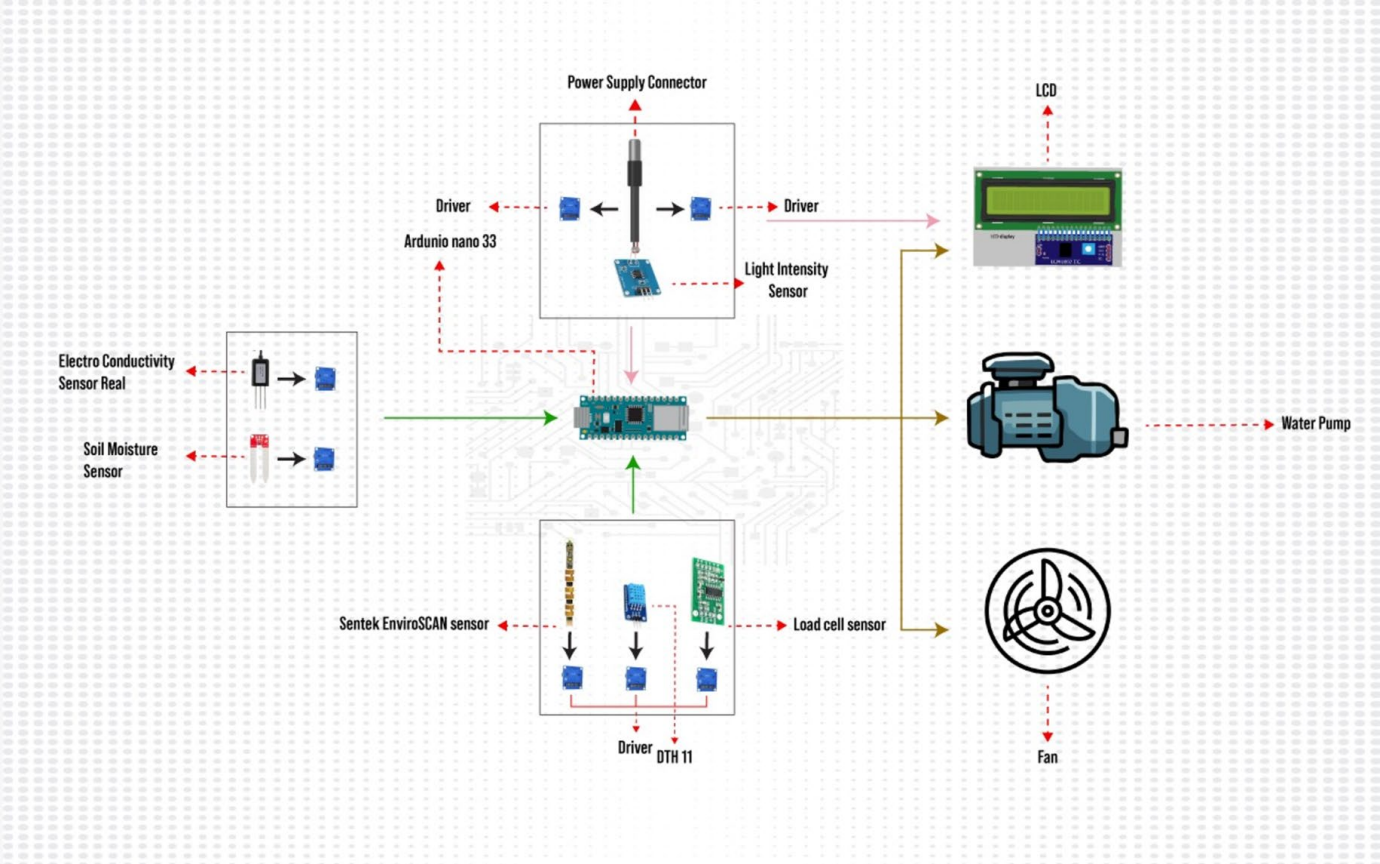
Circuitry diagram showing connection between sensors MCU and numerous devices.

Figure 2 represents the simulation circuit diagram showing the connection among various sensors, devices, and Arduino Nano 33. The sensors were selected based on the threshold values for optimum growth of saffron crops in the greenhouse. Temperature and humidity were monitored by using DHT-11 sensors, even though other sensors offered greater precision DHT-11 was more cost-effective and had a suitable range for multiple variables like temperature, soil moisture, light intensity, and PH level.

Secondly, we are cultivating saffron in non-fertilizer-based soil, and the optimal temperature range for saffron planting is between 20 to 30 °C. The DHT-11 sensor meets this requirement and is provided at a competitive range of price. Additionally, continuous real-time monitoring is essential for our research. The DHT-11 provides a sampling rate of 1 Hz, enabling us to obtain readings every second. In contrast, the DHT-22 has a slower sampling rate of 0.5 Hz, resulting in one reading every two seconds. Therefore, the DHT-11 is more suitable when real-time values are required. Lastly, considering power consumption, the DHT-11 consumes 0.3A of supply current, while the DHT-22 consumes 1A. This lower power consumption of the DHT-11 further supports its suitability for our research.

For pH, a pH sensor (pH 2.0 Interface) was selected due to its precision, sampling rate, and size. The greenhouse chamber is air sealed to facilitate the cultivation process at an average room temperature of 20 °C to meet necessary requirements such as air, temperature, humidity etc., and pH sensor is placed at the center of the greenhouse chamber for better calibration process, at the start the soil is completely covered in water to measure the initial readings of water level in soil once the reading is at 4.0 it is ready for measuring the real-time reading of water level in soil, In our research the level of pH in the soil is set at 6.0–7.0 which is an ideal value if the soil water level drops below 6.0 then the water pump is integrated to complete the water level to ensure the required amount of moisturizer for the cultivation of saffron. Saffron growth is also affected by the corm size so, a load cell sensor was used to measure the weight of corms. Load cell sensor readings are easily influenced by temperature and humidity, vibrations, and cross-talk when multiple sensors are integrated. To avoid such issues the load sensor is positioned outside the farming area in a dry location to ensure that humidity and temperature do not affect the seeds. Additionally, a metal plate measuring 4 × 4 cm is placed on top of the load sensor to stabilize its surface. The scale of the load sensor is then adjusted to ± 0 before testing the seed's weight. This adjustment helps prevent fluctuations in the weight readings, as vibrations or changes in the scale's calibration can affect the accuracy of the weight measurements. By ensuring the scale is properly zeroed before weighing, we can obtain precise and accurate weight measurements. Although the load sensor is slightly expensive it provides a high accuracy rate. Similarly keeping the threshold values in mind other sensors including a Light Intensity sensor and soil moisture sensor were selected to measure the intensity of light and moisture level for the saffron crop. Moreover, for measuring the soil salinity the Electric Conductive Sensors were used in the system to control and monitor the agronomical variables in saffron growth.

The industrial-grade pH sensor (pH 2.0 Interface) is utilized to maintain the optimal pH value of 6–7. The weight of the corm is measured with a load cell sensor with an operating voltage of 5–2 V with a precision value of 0.05%. The corm size is one of the most effective parameters for enhancing the saffron yield therefore accuracy in measurements is important, which can be obtained through a load cell sensor.

Sentek EnviroSCAN sensor is used to measure the volumetric water content as well as soil salinity. The Sentek EnviroSCAN sensor is a sophisticated tool designed for simultaneous measurement of soil moisture and salinity, crucial factors in agricultural settings like saffron cultivation. Employing Time Domain Reflectometry (TDR) technology, the sensor sends electromagnetic pulses through the soil via inserted probes. By analyzing the time taken for these pulses to travel and reflect back, the sensor calculates both soil moisture content and electrical conductivity, indicative of soil salinity. To ensure accurate measurements, the sensor is calibrated, aligning the parameters derived from pulse timing with actual moisture and salinity levels in the soil. This calibration process is vital for mitigating potential interference between the two measurements. While soil moisture and salinity are related, they can be influenced by different factors. To address this, the sensor applies advanced algorithms and signal processing techniques, allowing it to discern and separate the effects of moisture and salinity accurately. Additionally, the sensor's calibration and monitoring protocols can be customized for specific agricultural contexts, considering factors such as soil type, irrigation practices, and crop requirements like salinity tolerance. This tailored approach ensures that interference between soil moisture and salinity measurements is effectively managed, supporting optimal growth and yield in saffron cultivation and other agricultural applications. The voltage required to operate this sensor is 8–29 V along with a precision range for measuring soil moisture ± 3% and ± 5% for soil salinity. To maintain light intensity in the proposed system for saffron yield we have used Light Dependent Resistor (LDR) Sensor with operating power 100 mW. Saffron cultivation required a specific range of light intensity for optimal growth, as it required a minimum of 150 mol m^−2^ to 200 mol m^−2^ with + − 5 mol m^−2^. As we have conducted this research in a closed environment in a greenhouse chamber not in the open field after the natural daylight ends LDR sensor monitors simultaneously and an LED bulb is on to provide the light for the required amount. Saffron yield needs light for approximately 12–14 h so artificial lights are used to provide light to the saffron for specified hours. Various relays are used to control devices such as water pumps, fans, artificial lights, etc. Double channel relays are used to connect the water pump and fan that provide a medium between the Sentek EnviroSCAN sensor and PH sensor whenever the reading of any of the sensors goes below it generates the signal to relay, as the water pump starts pouring water in the soil and at the time when water is pouring humidity is increase and temperature is risen up for that fan is activated to reduce the temperature as this is their working principle in the IoT based system of greenhouse for saffron cultivation.

We have selected the microcontroller Arduino Nano 33 which has built-in Wi-Fi and Bluetooth connectivity. In our proposed system all devices are integrated with each other. We have used mobile broadband internet services for connecting Blynk IoT app and Arduino Nano 33 to transfer data of sensors over the internet because real-time data of the saffron greenhouse is held by the Arduino Nano 33 temporarily and must be sent to Blynk IoT app where it is saved permanently for future analysis and monitoring purposes. If there is disruption in the WiFi services at any point, the real-time monitoring will also be affected. In order to avoid this disruption mobile broadband services will be utilized as these are high speed and chances for errors are negligible. It allows to communicate wirelessly with various sensors and devices involved in the proposed system. The recommended voltage for operating Arduino Nano 33 is 3.3 Volts which is suitable for the proposed system. The Arduino Nano 33 microcontroller has 256 kB of flash memory for storing code and program data. It also has 32 kB of SRAM for storing variables and runtime data during program execution. The microcontroller does not have a separate ROM, and instead, its program code is stored in the flash memory. In order to address this storage issue in the Arduino Nano 33 microcontroller we have used Blynk IoT web/mobile app to store the real-time data until the farm owner ends the monitoring halting all the devices of the greenhouse system. The data stored in Blynk web/mobile app is permanently stored device or cloud or on the desktop of any computer that is being used for monitoring the saffron. The Arduino Nano 33 can be programmed using the Arduino Integrated Development Environment (IDE) and supports the Arduino programming language, which is based on C++. With these specifications, the Arduino Nano 33 is suitable for our system. The Arduino Nano 33 IoT has 22 digital input/output pins, including 6 analog input pins. These pins are used to connect numerous hardware devices as well as sensors to ensure their automated functioning. Two DHT11 sensors are used to measure the humidity and temperature, and they are connected to the Arduino Nano 33. By comparing both temperatures with threshold values stored in the database, actions like turning on the fan or heater can be controlled.

Since our IoT-based system for optimal saffron cultivation consists of various hardware devices as well as real-time communication failure could happen at any time. To prevent unexpected failures we have implemented several strategies for handling potential hardware failures including communication and hardware component failures etc. We are continuously monitoring the real-time values of different agronomical variables of the greenhouse sensed by various sensors. If the sensed values deviate from the normal threshold value, or if null or NAN values occur, we can easily guess there is some anomaly we can identify the faulty device through its id as all devices included in this system have unique identifiers. In addition to this approach, another feature is also integrated into the system which is an alarm system that alerts the farm owner via SMS alerts. All hardware components are modular and easily swappable which allows quick replacement of faulty devices without disrupting the entire system. A retry mechanism is also implemented in the system for communication failure which is best for error logging and debugging purposes.

In order to tackle the power outage problem in our greenhouse we have set up a power supply using an 18-V adapter connected to a battery backup. This backup helped us overcome power failure issues and ensured the continuous working system. To address software and communication failures, we rely on mobile broadband. Unlike regular internet traffic, mobile broadband is dedicated to internet use only, ensuring a consistent and uninterrupted connection. As a result, our sensor data updates in real-time. We have also fine-tuned the sensor settings to an ideal level, creating an environment where values and error rates remain minimal.

### Block and architectural diagram

**Figure 3:**
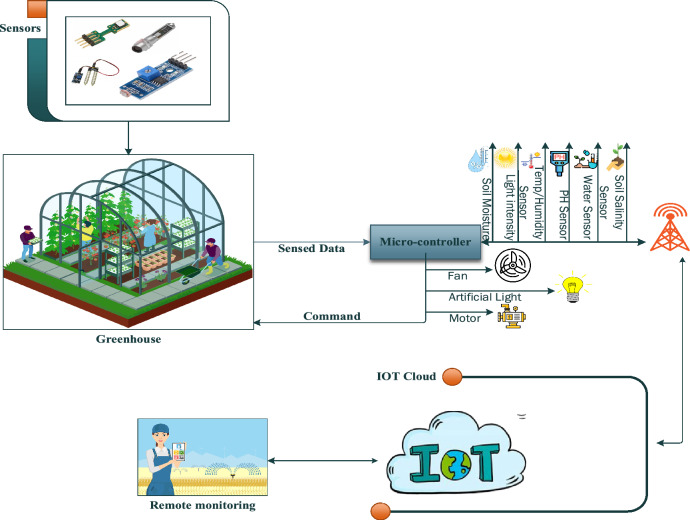
Block diagram for IOT based greenhouse for monitoring and controlling saffron.

Various factors affecting saffron growth include corm size, climate change, temperature, light intensity, soil moisture level, soil salinity, pH level, and suitable water availability at different stages which cannot be controlled in traditional farming. The proposed system is designed to control these agronomical variables to maximize the growth of saffron in IoT based greenhouse. Figure 3 presents the block diagram of the proposed system. The block diagram consists of two Arduino Nano 33 IoT MCU units and various sensors. Sensors are responsible for sensing agronomical variables. These sensors are capable of gathering real-time data on saffron yield from the greenhouse, and sending the data from the greenhouse to the microcontroller (Arduino Nano 33 IoT) for decision-making. For decision-making purposes sensed data is compared with the data saved in the database, and updates are sent to the farm owner via an application.

**Figure 4:**
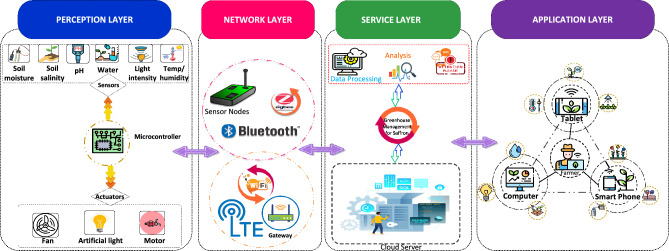
Architectural diagram of IOT based greenhouse for saffron.

Figure 4 presents the layered architecture of a proposed system that consists of four basic layers namely the perception layer, network layer, service layer, and access layer.

i. Perception layer: This layer is also referred to as the physical layer. This layer consists of all sensors involved in the proposed system, microcontrollers, and actuators for controlling and managing agronomical variables involved in the greenhouse. These sensors and actuators include temperature sensors (DHT11) are used to measure the temperature and humidity, Load cell sensors for measuring the weight of the corm, SentakEnviroSCAN for measuring and LDR and actuators including water pump, heating and cooling system (Fans), LED lights. All objects presented on this layer are responsible for monitoring, data collection, and transferring data from the Greenhouse environment to the Arduino nano 33 microcontrollers where data is stored temporarily for further analysis and decision-making.
ii. Network layer: Data transmission between different layers is the prime goal of our proposed system for making analysis and different decisions. The network layer is used for this purpose which consists of networking technologies to build connections between different layers for exchanging data. Data collected at the perception layer is sent to the network layer. The network layer is responsible for transmitting this sensed data to the service layer for IoT-based Greenhouse for saffron.
iii. Service layer: Data analysis is considered one of the most crucial parts of IoT-based systems. The service layer involves the analysis of agronomical variables collected from the Greenhouse and data processing. Careful analysis of various agronomical variables including temperature, humidity, soil moisture, pH level, humidity, and available water is done on this layer. Sensed data is compared with threshold values stored on a cloud server and corresponding alert messages will be sent to the farm owner for efficient management of the proposed system.
iv. Application layer: Provides an appropriate platform for the farmers to monitor and control his/her Greenhouse crop efficiently. The results of analyzed data from the service layer will be sent to the farmer via an application and a farmer will be able to check the information on his/her smartphone, tablet, or laptop and take action accordingly.

### Experiment and results

The experiment was conducted in the month of June 2023 under extreme weather conditions. External environmental conditions such as humidity, temperature, light intensity, and soil moisture were measured with the help of IoT based monitoring system. Parameters measured from the external environment act as boundary conditions for the greenhouse developed in this research to enhance saffron growth under unfavorable circumstances. The threshold values of saffron used in this experiment against mentioned agronomical factors are listed in Table 2. All these threshold values are collected from research studies^24,39,41–44,46–50,54,57,59,61–74^. The Placement of sensors within the greenhouse environment was determined through various considerations aimed to achieve accurate monitoring of soil and other various environmental factors. Ground-based sensors were positioned at a depth of 30–40 cm below the surface of the soil to come into the typical root zone where vital parameters like moisture, salinity, and pH greatly influence plant growth. This sensor placement strategy targets not only the plant root area but also shields the sensors from direct sunlight or physical damage that’s common in agricultural activities. Moreover, numerous sensors placed through the greenhouse provide various important readings from the greenhouse growing area. The environmental sensors such as temperature and humidity sensors were places at an optimal height of 40-50 cm above the ground in order to capture conditions at level of canopy where plant growth is greatly affected by factors as air temperature, humidity and light intensity and to ensure accurate monitoring of the plant growth while reducing the interference's that occur from other greenhouses throughout the process. Overall, the optimal and careful placements, its accessibility contributes to effective greenhouse management allowing the farmers or growers to make optimized decisions for crop yields.

**Table 2:** Threshold values of agronomical variables.

Agronomical variables	Optimum values
Corm size	9–10 g
Temperature	20–30 °C
pH level	6–7
Light intensity	200 (3R2B)
Light requirement	12–14 h
Salinity	2–3 dS m^−1^
Soil moisture	10–12%
Relative humidity	60–85%
Water availability	300–500 mm

One motor, two cooling fans, and 3 artificial lights are attached to the system. Soil taken for the greenhouse is loamy soil which is a mixture of sand, clay, and silt, enriched with nutrients and provides good drainage. Loamy soil is best known for its balanced composition of sand, silt, and clay particles with a pH level of 6–7.5 due to which it is a top-notch choice for saffron cultivation. The sand content present in loamy soil assures excellent drainage, preventing waterlogging and minimizing the risk of corm rot a universal issue in poorly drained soils. Similarly, the presence of silt and clay particles allows the loamy soil to keep the moisture effectively which in turn helps in providing steady water to the corm throughout. Moisture keeping is crucial for healthy growth and flower production. Furthermore, the nature of loamy soil is rich in nutrients, organic matter promotes the growth of the plant and its development. The loose and crumbly texture of loamy soil makes it easy to facilitate planting for saffron corms. Overall, its balanced characteristics create optimized conditions for saffron cultivation making it to help in supporting top-quality saffron production.

After preparing soil saffron blubs were planted into it with 10 cm gap between each blub. Specific planting techniques are employed in our experiment of saffron cultivation including planting depth and spacing between blubs. This planting technique has a great influence on the development and growth of saffron. The spacing between blubs is around 10 cm making sure that each corm has sufficient room to grow and expand as well as reducing the competition for required nutrients, sunlight, and water in neighboring plants thus promoting healthier and optimal saffron production. Consistent spacing between blubs ensures uniform distribution of resources by reducing over crowding for optimal saffron growth. Saffron blubs are planted at a depth of 10–15 cm below the soil to protect them from extreme temperatures, and provides stability while supporting health plant development. It also ensures that the emerging roots have enough soil around them to protect them from any damage thus contributing to the overall development and growth of the plant and strong roots. Planting the bulbs at appropriate places and depths helped in the experiment as it led to rapid growth and development of the plants. The variations observed in the growth of plants could possibly influence the outcome from various factors such as soil fertility, the condition of the surrounding environment, and practices of management. The variations we observed in plant spacing and its depth could potentially affect the overall nutrient availability, its overall growth, performance, and development thus decreasing its competition among other plants in the environment. The variations could also possibly arise from other environmental factors such as water retention, disease incidences, and soil temperature impacting the overall health and growth of saffron. Overall, effective techniques and methods are essential for optimizing saffron cultivation, its overall growth, and getting the desired outputs. By keeping all these points in mind careful consideration for planting saffron blubs was taken in the experiment setting.

**Figure 5:**
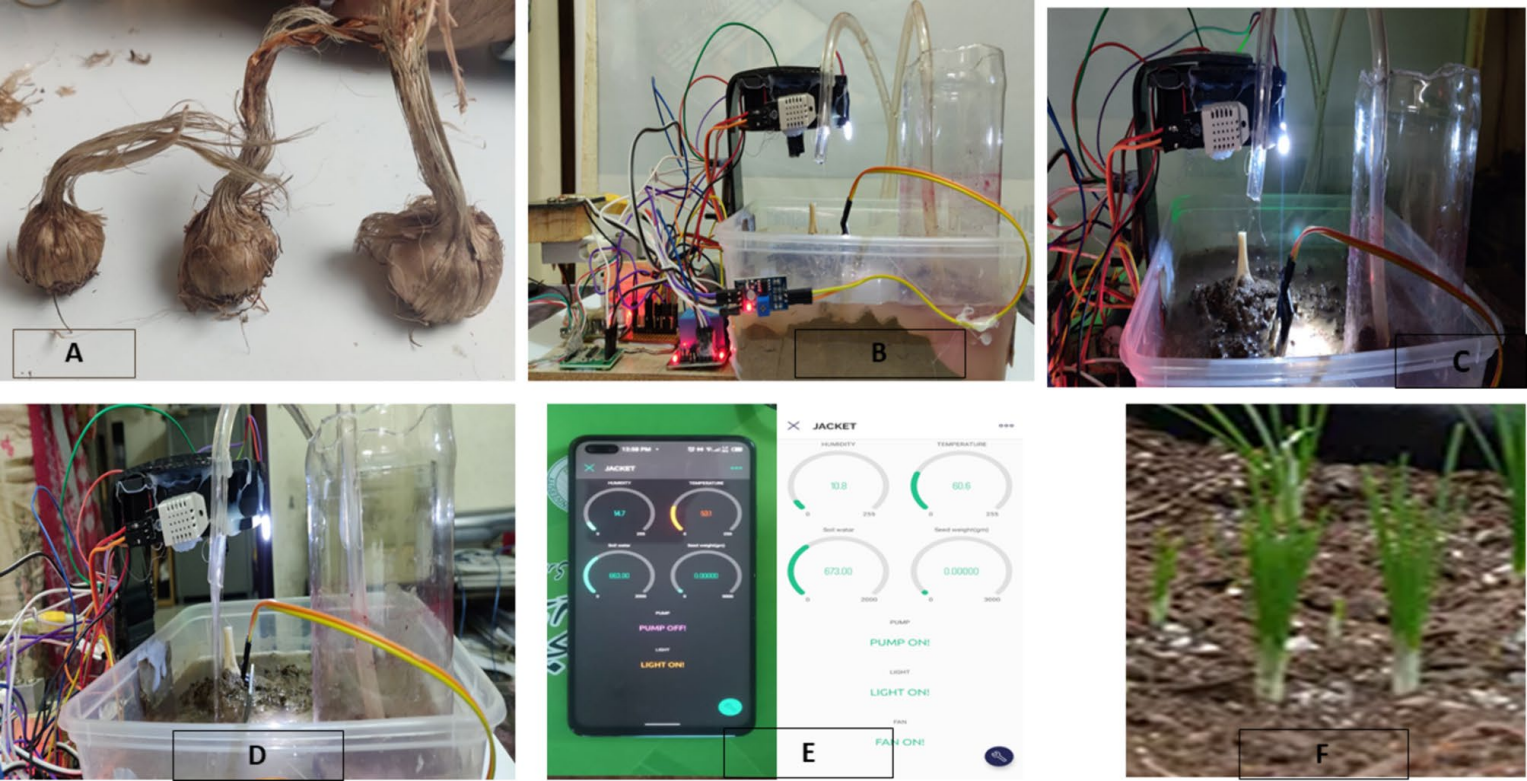
(**A**) Saffron corms; (**B**) hardware components for greenhouse; (**C**) complete setup of greenhouse; (**D**) greenhouse system with pump on; (**E**) notification app for greenhouse; (**F**) saffron sprouting.

After sowing the blubs carefully sufficient amount of water was provided. The greenhouse designed in this research for maximizing the growth of saffron is shown in Fig. 5 which consists of various phases of the system. In Fig. 5 point A shows the saffron blubs used in the experiments. These blubs are planted with appropriate planting techniques including optimal depth and spacing. B shows the hardware setup of the developed system, in this phase various sensors microcontrollers, and actuators are incorporated for automatic monitoring of numerous environmental factors such as temperature, humidity, pH, soil moisture, soil salinity, and light intensity. All these sensors sense the real-time data of the saffron field and send it to the microcontroller. Point C presents the complete system setup in running form with light actuator status to stimulate the optimal lightening conditions for saffron blubs to grow. Moreover, D point shows another variation of the system where the placement of sensors and different actuators, and point E depicts the monitoring app for the current system called the Blynk IoT app. The Blynk IoT app plays a critical role in the monitoring and management of our greenhouse system for optimal saffron cultivation. With its user-friendly and easy-to-use dashboard, the farmers can easily monitor real-time data of numerous environmental factors such as temperature, humidity, soil moisture, pH, soil salinity, and light intensity. This remote real-time monitoring allows farmers to keep track of optimal growth conditions. In case of any deviation from the desired condition farmer is notified via customized alerts and notifications enabling prompt actions to mitigate risks and maintain optimal growth conditions. Moreover, the app's data logging and analysis features offer valuable insights into saffron cultivation trends, facilitating informed decision-making to optimize crop yields and quality. Additionally, Blynk IoT app allows us to create a folder in the phone directory to store specific real-time values of the saffron field for future analysis. Moreover, Overall, the Blynk IoT app provides farmers with a comprehensive toolset for efficient monitoring and management of greenhouse systems, ultimately contributing to improved saffron cultivation outcomes. Last but not least Point F is the final stage where the growth of saffron in the greenhouse is presented.

### Operational framework and hardware synergy

**Figure 6:**
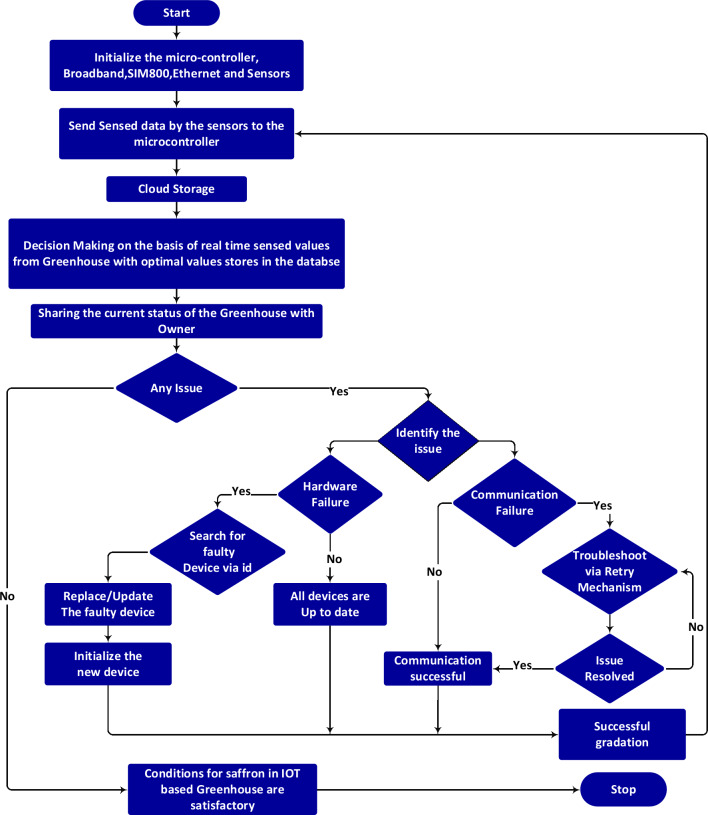
Flow chart diagram of IOT based greenhouse for saffron.

Figure 6 shows the workflow of the proposed system. The sensors deployed analyze various agronomical variables such as temperature, corm size, pH, soil moisture, humidity, soil salinity, light intensity, and light requirements at short intervals, and the data is stored in the cloud. The real-time sharing of data is made available on mobile and laptop interfaces for predictive analysis. Since real-time communication is taking place over the internet data security is crucial in such cases. We established a single admin user login to overcome security concerns by centralizing control to access and manage data. As the system contains crucial information about saffron cultivation it is mandatory to give access to only authentic users to keep data integrity. We have added an extra layer of security by adding verification code ensuring more data security and integrity. Additionally, the email is more suitable such as G suite, Microsoft, or Yahoo are integrated for farmers’ login to enhance security. By using existing email and passwords user can securely access the Blynk Iot app from their laptops or mobile device. After successful login user can navigate to the app and monitor the readings of various agronomical values make informed decisions and analyze the data of various days. First Microcontroller and sensors will be initialized. Weight (> 8 g) of saffron corm will be checked first by using a load cell sensor. After initialization, the values sensed by the sensors namely temperature, humidity, soil moisture, Light intensity, Soil salinity, and pH will be sent to the microcontroller. Arduino Nano 33 IoT microcontroller will transmit these values to the database where real-time sensed values will be compared with optimal values for these agronomical factors saved. DHT11 sensor will sense the temperature of the greenhouse environment. When the temperature of the greenhouse for the saffron crop exceeds the limit of the threshold value, the system will turn on the fan automatically. When the temperature of the greenhouse becomes normal fan will be turned off automatically. Farmer can monitor all these activities on his/her mobile phone or laptop. Similarly, when the sensed value of real-time humidity crosses the threshold value of the saffron, a message will be sent to the user on his/her mobile, and in return exhaust fan will be turned on automatically. After getting a normal range of humidity level in the greenhouse the exhaust fan will be turned off automatically. The light intensity sensor will check the threshold value of light intensity for saffron if it crosses the limit then artificial lights will be turned on otherwise artificial lights will stay off. In case the pH value of greenhouse soil for saffron crosses the defined limit a message will be sent to the farmer and appropriate action will be taken automatically. If soil moisture deficiency is sensed by the sensor the sensed value will be sent to the user along with a deficiency message and the microcontroller will automatically turn on the water pump. Once the moisture level reaches its desired level the water pump will automatically be turned off and a message will be sent to the user. After getting enough water the sensor will again send the updated values to the controller which will be transferred to the Blynk IoT app for analysis purposes.

During this phase, we faced several challenges in sensor synchronization where accurate data was the major concern. Variations in two different sensor readings could potentially affect the precision of our proposed system. This challenge was addressed by implementing extensive calibration, and sensor placement, and the distance between them was increased to minimize the interference and ensure the data consistency. Load cell sensors were first inside the farming area but due to interference of several factors like vibrations, temperature the sensors were not working properly to resolve this issue load cell sensors were placed outside the farming area in a dry place, and a metal plate measuring 4 × 4 cm is placed on top of the load sensor to stabilize its surface.

In our IoT-based system for optimal saffron cultivation, accurate sensor readings in real-time are very crucial. We have identified the most essential agronomical variable that plays a vital role in optimal saffron growth like soil moisture, temperature, light intensity, soil salinity, and humidity, and after that appropriate sensors were selected. Ground truth data is collected manually which serves as a benchmark for sensor calibration. We have positioned sensors like a fan, LED blubs, and water pumps in a center-aligned manner to ensure that all areas of the Greenhouse get the required intensity of light and airflow. The ground base sensors with a certain depth and distance are placed for the accurate monitoring of the complete saffron filed. Additionally, calibration is conducted over the internet, we have adjusted the minimum required values by reducing them by 0.5, and have tolerance rate is set to ± 5/0.5 as per sensor range.

Table 3 presents the comparative analysis between different research articles based on various agronomical variables and technologies used. In this comparative analysis, we have set numerous agronomical variables and technologies as evaluation criteria. Various research studies conducted for optimizing saffron growth in fields and controlled environments like greenhouses are compared with our IoT-based system for greenhouses. Saffron is climate sensitive crop and its growth is affected by various environmental factors such as temperature, humidity, pH, soil salinity, soil moisture level, light intensity water availability for this reason we have chosen these factors as evaluation criteria along with suitable sensor technologies for controlling and monitoring our system automatically by reducing labor cost and managing resources efficiently and promoting sustainable agriculture. Our IoT-based system excels in different aspects as compared to existing work by using superior sensor integration and monitoring the most crucial environmental factors in real-time in a controlled environment which is not possible in the open field. This new IOT-based system has the potential to optimize the saffron growth in the greenhouse in an efficient manner.

**Table 3:** Comparison analysis of technologies used in various articles.

Ref/year	IOT	saffron	Cloud computing	Greenhouse	Agronomical variables									Application domain		Sensors						
					Corm size	Temperature	pH	Light intensity	Light Req	Salinity	Soil moisture	Humidity	Water availability	Monitoring	Controlling	Temp/humidity	pH	Light intensity	Soil moisture	Water	Soil salinity	Load cell
^39^	✓	✓		✗	✓	✓	✓	✗	✗	✗	✗	✓	✓	✓	✓	✓	✓	✗	✗	✓	✗	✓
^44^	✗	✓	✗	✗	✗	✗	✓	✗	✗	✓	✗	✗	✗	✗	✗	✗	✗	✗	✗	✗	✗	✗
^63^	✗	✓	✗	✗	✗	✗	✗	✗	✗	✓	✗	✗	✗	✗	✗	✓	✗	✗	✗	✗	✗	✗
^64^	✓	✓	✗	✗	✓	✓	✓	✗	✓	✗	✗	✓	✗	✗	✗	✓	✓	✗	✗		✗	✓
^61^	✓	✓	✓	✗	✗	✗	✗	✗	✗	✗	✗	✗	✗	✓	✓	✓	✓	✓	✓	✗	✗	✗
^59^	✗	✓	✗	✗	✗	✗	✓	✗	✗	✓	✗	✗	✗	✗	✗	✗	✗	✗	✗	✗	✗	✗
^62^	✗	✓	✗	✗	✗	✗	✓	✗	✗	✓	✗	✗	✗	✗	✗	✗	✗	✗	✗	✗	✗	✗
^65^	✓	✓	✗	✗	✗	✓	✓	✗	✗	✗	✗	✓	✓	✓	✗	✓	✗	✗	✗	✓		✓
Proposed	✓	✓	✓	✓	✓	✓	✓	✓	✓	✓	✓	✓	✓	✓	✓	✓	✓	✓	✓	✓	✓	✓

### Scalability and adaptability of proposed system

Addressing the scalability and adaptability of the proposed IoT-based system for saffron cultivation involves several key strategies to ensure it can be effectively applied across various greenhouse sizes, environmental conditions, and crop requirements. The system follows a modular approach, allowing for easy expansion or reduction based on the size of the greenhouse and the volume of crop production. This modularity extends to sensors and control units, which can be added or removed to suit the scale of operation and the specific needs of the saffron crop being cultivated. To accommodate variations in environmental conditions and crop requirements, the system incorporates sensors that have the capability to adjust operational parameters based on real-time data.

Interoperability with existing agricultural management systems is a critical consideration in the development of our system. The system's architecture is designed to be flexible, supporting customization to meet the unique requirements of different agricultural practices and environmental conditions. This adaptability ensures that the system remains relevant and effective as farming practices evolve and new challenges emerge in the agriculture sector.

### Analysis of threshold deviation

During the course of our experiment with the IoT-based system for saffron cultivation, there were indeed instances where the measured values of agronomical variables deviated significantly from the set threshold values. Such deviations are not uncommon, given the sensitivity of saffron to environmental conditions and the variability of these conditions over time. Whenever these deviations occurred, the system's automated control mechanisms were triggered to address the discrepancies and restore optimal growing conditions. For example, the temperature readings exceeded the optimal range in June for saffron growth, system activated the cooling mechanism to reduce the temperature. Additionally, the system is equipped to adjust the humidity and pH levels automatically, based on real-time data, to ensure they remain within the optimal ranges for saffron cultivation. Keeping in view the significant deviations prompted a review of the system's calibration and the accuracy of its sensors. In some cases, corrective actions included recalibrating sensors or replacing faulty units to ensure reliable data collection. These steps were crucial for maintaining the integrity of the system and its ability to provide precise control over the greenhouse environment.

### Flow chart diagram

Sensors are continuously monitoring and controlling environmental factors such as temperature, pH, humidity, soil moisture, and salinity as well as light intensity in the greenhouse. The real-time sensed data is further sent to the microcontroller. It acts as a central processing unit for receiving and analyzing sensed data. This data is then further sent to the cloud for permanent storage. Farmers monitor the data regularly via Blynk IoT app for analysis. A feedback loop is incorporated in the system where the microcontroller manages the environmental factors based on sensed and threshold values. If any sensed value is above or below its optimal range microcontroller will control that factor by activating its specific actuator making sure that after adjustment variables are within the optimal range.

The current system uses two different techniques for checking errors. Firstly while monitoring if the farmer finds any unusual pattern in stored data they will identify the error type whether the error is caused by the hardware device or it is due to communication failure. If the error is hardware-based then search for the faulty device using its unique identifier. Fix/ replace the faulty device and initialize the new device. Moreover, if the error is based on communication failure troubleshoot to resolve the error via retry mechanism. If an error is resolved go ahead otherwise troubleshoot again until the issue is not resolved. Regular monitoring ensures that all system components are working properly and incase of any error alerts for generated that require maintenance.

### Optimal condition management technique

The system is continuously monitoring various environmental factors through sensors. When any of the variables deviate from their optimal range, the system instantly identifies this through real-time data analysis. In scenarios where multiple variables reach critical levels at the same time, the system employs an algorithm to prioritize responses based on urgency and potential impact on saffron health and yield. For example, if both temperature and soil moisture levels are outside their ideal ranges, but the temperature poses a more immediate threat to crop viability, the system will prioritize temperature regulation actions. Once priorities are established, the system automatically executes corrective actions. This could involve adjusting irrigation systems, activating heating or cooling mechanisms, or altering the greenhouse's ventilation to address the identified issues promptly. After implementing the corrective actions, the system continues to monitor the affected variables closely to ensure that the adjustments have had the desired effect. Throughout this process, the system keeps farm owners informed by sending real-time data and alerts to their devices through Blynk IoT. This ensures that the owners are aware of any critical deviations and the system's responses, allowing them to intervene manually if desired.

### Ethical statement

Authors consciously assure that for the manuscript “Internet of Things (IoT) Based Saffron Cultivation System in Greenhouse” the following is fulfilled:

1. This material is the authors' own original work, which has not been previously published elsewhere.
2. The paper is not currently being considered for publication elsewhere.
3. The paper reflects the authors' own research and analysis in a truthful and complete manner.
4. The paper properly credits the meaningful contributions of co-authors.
5. The results are appropriately placed in the context of prior and existing research.
6. All sources used are properly disclosed (correct citation). Literally copying of text must be indicated as such by using quotation marks and giving proper reference.
7. All authors have been personally and actively involved in substantial work leading to the paper, and will take public responsibility for its content.

The violation of the Ethical Statement rules may result in severe consequences.

I agree with the above statements and declare that this submission follows the policies as outlined in the Guide for Authors and in the Ethical Statement.

## Conclusion

In this article, we have proposed an IoT based greenhouse system for optimizing the production of saffron globally. To accomplish this goal different agronomical variables playing a crucial role in saffron growth such as temperature, humidity, soil salinity, soil moisture, pH, light intensity, and requirements were investigated through literature. We have defined four layers for our new system where each layer is responsible for executing a specific task. The perception layer deals with all the physical devices involved in the system including sensors, microcontrollers, and actuators. The physical objects present at the perception layer are accountable for the collection of real world data from the greenhouse sensed by the sensors and sending it to the microcontroller for decision making. All the sensed data from the perception layer is sent to the network layer which has different network topologies with the help of which connection is built between various layers of the system. Whereas on the service layer of the system, careful analysis of agronomical variables discussed in this work is done by comparing the sensed values with the threshold values. Farmers can view and get notified about his/her greenhouse crop via messages for effective management of the saffron. We have defined the application layer for our system as well which acts as a bridge between farm owner and greenhouse. The experiment results showed that the proposed system has the potential to maximize saffron production in the greenhouse by controlling crucial agronomical variables. Furthermore from the literature, it can be seen that no such system exists for optimizing saffron growth in soil base greenhouse. This will be the first system mainly focusing on the most promising agronomical factors affecting the growth of saffron in traditional open-field farming. This system will also help the farmers to remotely monitor their farms anytime from anywhere with the help of web or mobile applications.

### Future work

Given the dynamic nature of saffron cultivation and the rapid advancements in IoT and agricultural technologies, the future developments of our proposed IoT-based system aim to further enhance greenhouse management and crop production by addressing emerging challenges and leveraging new opportunities. We envision incorporating advanced machine learning algorithms and AI to better predict environmental changes as well as pest detection and control systems to optimize crop growth conditions in real-time, thus increasing the system's adaptability and precision. Integration of cutting-edge sensors for more granular monitoring of environmental factors will also be essential, enabling the detection of subtler changes in the crop's microclimate that could impact growth.

Machine learning or predictive analytics algorithms can play an essential role in enhancing the predictive capabilities and cultivation practices of our IoT-based greenhouse system. By leveraging historical data and real-time data recorded by the sensors against several environmental factors can be used to train different machine learning algorithms to predict saffron growth patterns, and future environmental conditions more accurately. Moreover, these algorithms can also be trained in historical data recorded by the sensors against several environmental factors such as temperature, humidity, soil salinity moisture level and light intensity to find the correlation between these factors and their effect on saffron growth. Secondly by analyzing real-time data streams coming from different sensors ML classifiers can be used to detect anomalies or changes in expected patterns for efficient management. With the help of machine learning, we can also enable predictive maintenance of our hardware equipment with in the greenhouse.

## Data Availability

The dataset available publically on GitHub: https://github.com/RabiaKhan-94/ECOFARMING.
